# StreaMD: the toolkit for high-throughput molecular dynamics simulations

**DOI:** 10.1186/s13321-024-00918-w

**Published:** 2024-11-05

**Authors:** Aleksandra Ivanova, Olena Mokshyna, Pavel Polishchuk

**Affiliations:** 1 Institute of Molecular and Translational Medicine, Faculty of Medicine and Dentistry, Palacky University, Hnevotinska 5, 77900 Olomouc, Czech Republic; 2 Institute of Organic Chemistry and Biochemistry of the Czech Academy of Sciences, Flemingovo náměstí 542/2, 160 00 Praha 6, Czech Republic

**Keywords:** Molecular dynamics, High-throughput molecular dynamics, Distributed simulations, GROMACS

## Abstract

Molecular dynamics simulations serve as a prevalent approach for investigating the dynamic behaviour of proteins and protein–ligand complexes. Due to its versatility and speed, GROMACS stands out as a commonly utilized software platform for executing molecular dynamics simulations. However, its effective utilization requires substantial expertise in configuring, executing, and interpreting molecular dynamics trajectories. Existing automation tools are constrained in their capability to conduct simulations for large sets of compounds with minimal user intervention, or in their ability to distribute simulations across multiple servers. To address these challenges, we developed a Python-based tool that streamlines all phases of molecular dynamics simulations, encompassing preparation, execution, and analysis. This tool minimizes the required knowledge for users engaging in molecular dynamics simulations and can efficiently operate across multiple servers within a network or a cluster. Notably, the tool not only automates trajectory simulation but also facilitates the computation of free binding energies for protein–ligand complexes and generates interaction fingerprints across the trajectory. Our study demonstrated the applicability of this tool on several benchmark datasets. Additionally, we provided recommendations for end-users to effectively utilize the tool.

**Scientific contribution**

The developed tool, StreaMD, is applicable to different systems (proteins, ligands and their complexes including co-factors) and requires a little user knowledge to setup and run molecular dynamics simulations. Other features of StreaMD are seamless integration with calculation of MM-GBSA/PBSA binding free energies and protein-ligand interaction fingerprints, and running of simulations within distributed environments. All these will facilitate routine and massive molecular dynamics simulations.

**Supplementary Information:**

The online version contains supplementary material available at 10.1186/s13321-024-00918-w.

## Introduction

Molecular dynamics (MD) simulations and the computation of binding free energies represent pivotal methodologies within computational chemistry and molecular biology [1–3]. MD simulations facilitate the exploration of atomic and molecular motion and study of intermolecular interactions. Concurrently, the calculation of binding free energies associated with ligand–protein interactions can unveil the most plausible binding modes by virtue of ranking docking poses [4–6]. Moreover, it enables the prioritization of compounds for subsequent experimental evaluation by ranking ligands [4, 7]. This capacity to discern binding affinities and interactions has become increasingly pivotal in contemporary structure-based virtual screening pipelines, owing to the expanding availability of high-performance computing resources, thereby establishing the calculation of binding free energies as an integral component of such workflows.

The setup of MD simulations and the computation of binding free energies demands a certain level of expertise and knowledge. This process can be susceptible to errors (e.g. setting up force field, solvent box, simulation parameters, etc.) when executed manually, especially when dealing with multiple ligands and complexes. Structure preparation necessitates a series of steps, each requiring careful parameter selection to yield valid results. Consequently, the automation of these intricate procedures and the development of simplified, user-friendly pipelines for MD simulations and free energy calculations are imperative to facilitate structure-based virtual screening pipelines and enable the easy assessment of hundreds or thousands of ligands within a single screening campaign.

Several endeavors have been made to streamline MD protocols for end-users, thereby reducing the demand for specialized knowledge in the domain of molecular simulations. We are not going to address here multiple existing in-house solutions to MD automation due to their inaccessibility to the wider scientific community. Among accessible solutions, OpenMM, for instance, provides a versatile framework for constructing customized pipelines for MD simulations [8]. Building upon OpenMM, the OpenMMDL tool (available at https://github.com/wolberlab/OpenMMDL) has been designed to simplify the preparation of protein and ligand structures for MD simulations. It offers a web-based interface to generate a set of scripts using input files, facilitating the execution of MD simulations. Similarly, CharmmGUI [9], a widely recognized web-based platform, generates scripts encompassing all simulation steps, which must then be executed by the user. CharmmGUI offers a comprehensive set of features, including membrane system preparation, support for various MD software, and even high-throughput structure preparation. However, users must still manually manage all steps, including waiting times between preparation stages. Ultimately, users are responsible for executing the generated scripts and must design their own pipeline if they wish to perform multiple simulations in a distributed computing environment. Tools like HTMD [10] and ACEMD [11] enable the creation of customized pipelines and the execution of MD simulations on single servers and clusters. However, these tools require the development of tailored pipelines suitable for processing multiple protein–ligand complexes in a single execution. Galaxy is the data analysis platform, which incorporates multiple tools (including MD) and provides a web-based interface to execute MD simulations within a distributed environment [12]. A notable advantage lies in the ability to perform MD simulations involving multiple ligands bound to the same protein target through a straightforward process. However, this necessitates the installation and configuration of the tool on a cluster. Other difficulties may arise with cofactor-dependent system simulations or automatic continuation of interrupted runs since the default workflows do not support such functionalities. Also the tool does not support so far Gaussian and MCPB.py parametrization. Recent developments include Uni-GBSA [13] and ChemFlow [14], both primarily focused on the calculation of binding free energies using the MM-GBSA/PBSA approaches and the implementation of simplified, user-friendly pipelines. While Uni-GBSA supports not only the calculation of binding free energies but also conventional MD simulations of proteins or protein–ligand complexes, it is not inherently designed for high-throughput simulations, requiring users to establish their own pipelines for execution in a distributed environment. On the other hand, ChemFlow can be executed on distributed systems operating under SLURM or PBS schedulers, but its ScoreFlow module is primarily geared towards the re-scoring of docking poses using the MM-GBSA/PBSA approaches and is not very suitable for conventional MD simulations. Hence, an evident gap persists in the availability of tools that can automate the most common MD simulations and are amenable to execution on distributed systems without necessitating specialized knowledge in their operation.

We have established an automated pipeline designed to facilitate explicit-solvent MD simulations across various systems, including proteins, protein-cofactors, protein–ligand complexes, and protein–ligand-cofactors systems. Notably, our pipeline distinguishes itself by accommodating simulations involving cofactors, which are often intrinsic components of proteins and are of critical significance for obtaining accurate simulation results. The key feature of this pipeline lies in its comprehensive automation, encompassing all stages of the simulation workflow, commencing from system preparation and extending through to the execution of production simulations.

It is noteworthy that our developed pipeline seamlessly supports systems necessitating customized atom types and force fields, such as cases involving specific metal ions within a binding site or ligands containing boron atoms. Importantly, this support is integrated and does not impose additional burdens on the user. Furthermore, our tool permits the easy continuation or extension of simulations as required.

Additionally, we have integrated MD simulation pipelines with the computation of binding free energies utilizing the MM-GBSA/PBSA methodology and the analysis of protein–ligand contacts. These simulations and calculations can be executed on both single servers and distributed systems. The incorporation of distributed systems has been achieved through the utilization of the Dask library, which removes the need for a dedicated scheduler and enables operation across a network of computers. This development empowers the performance of high-throughput MD simulations and the calculation of binding free energies for a substantial number of ligands, all achieved with minimal user efforts.

These features set StreaMD apart from other widely used tools such as CharmmGUI and OpenMMDL, which primarily automate the generation of scripts that users must execute manually. Consequently, users of CharmmGUI and OpenMMDL as well as Uni-GBSA are required to develop their own pipelines when performing MD simulations on a large scale in distributed environments. Galaxy is able to perform large scale simulations, however, there can be problems with systems containing cofactors and continuation of interrupted calculations, which were solved in StreaMD.

### Implementation

**Fig. 1:**
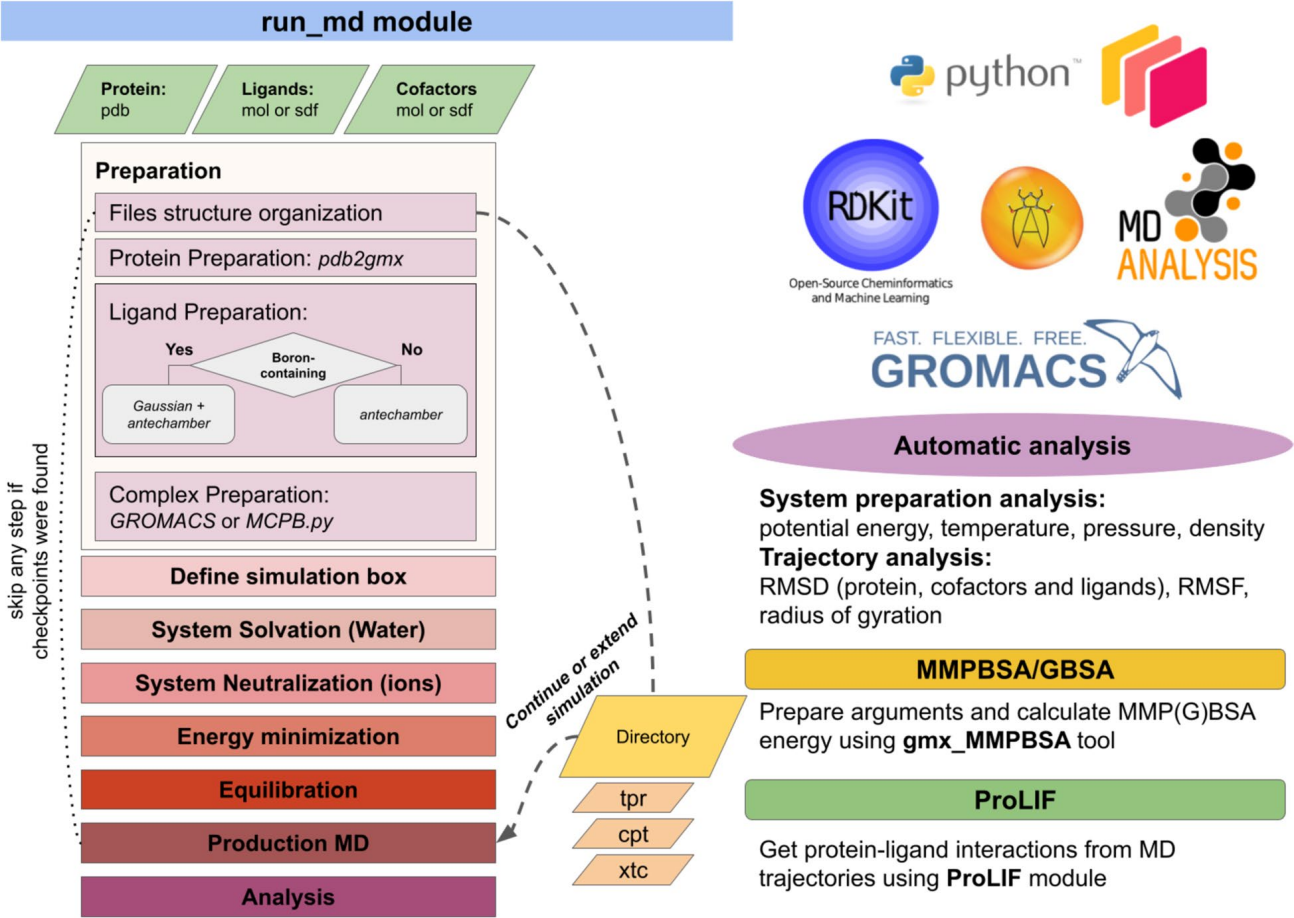
Overview of the StreaMD pipeline

The module has been implemented using Python 3 and is designed to operate within the UNIX operating system environment. Illustrated in Fig. 1 the general workflow delineates the operational sequence. Users are required to supply a prepared protein structure in PDB format, ensuring its completeness by addressing any missing residues and side chains, while also ensuring protonation and, in particular, explicitly setting histidine protonation states. Furthermore, users have the option to submit one or more ligands and/or cofactors in MOL or SDF formats, with coordinates aligned with those of the submitted protein.

The tool relies on a strict hierarchy of files and directories within the user specified output directory – the *root* directory. This directory structure will be created automatically upon running of corresponding simulations. All MD files will be stored in the *root/md_files* directory and all log files will be stored in the *root* directory directly. Files created during protein, ligand and cofactors preparation are stored in *root/md_files/md_preparation/{protein, ligands, cofactors}.* Complex preparation and production run MD files are stored into *root/md_files/md_run/${protein-name}_${ligand-id}/*. In the case if such directories already exist, the tool will search for checkpoint files to skip previously completed steps and will continue an interrupted run.

StreaMD offers two operational modes: conducting simulations and extending existing simulations. In the latter mode, users are required to submit either a directory containing the preceding run generated by StreaMD or external files in *tpr*, *cpt*, and *xtc* formats. Additionally, users must specify the desired extension duration for the simulation in nanoseconds.

Ligand and complex preparation stages, as well as MD simulation and subsequent analysis, are conducted individually for each submitted system (complex). All these tasks are parallelized based on CPU and GPU core allocation. Meanwhile, MD production simulations can be either parallelized on a per-node basis or by the --*mdrun_per_node* argument where allocated CPUs and GPUs will be distributed over multiple simulations. The user retaining the option to restrict the maximum number of CPU cores utilized per node. The parallel execution is facilitated through the utilization of the Dask library, which has previously demonstrated efficacy in our EasyDock tool for distributed docking [15]. Dask, a Python library tailored for parallel and distributed computing, supports execution across various clusters or a network of servers via SSH connections. To activate parallel processing, users are required to submit a text file containing the node addresses to be utilized by a Dask SSH cluster.

### Protein preparation

Before the start of simulations, a user should prepare the protein structure:

1. Complete missing residues and reconstruct missing loops
2. Resolve alternative residue locations
3. Remove co-crystallizated ligands and water molecules, if any
4. Protonate the protein at a chosen pH value
5. Check protonation states of amino acids, in particular for histidines to put proper aliases HIE, HID or HIP (otherwise protonation may be changed during MD preparation stage)

StreaMD provides automatic processing of the submitted protein structure by executing the command *gmx pdb2gmx*, which reads a pdb file, reassign hydrogens according to amino acid residue names and writes coordinates and a topology in GROMACS format. By default, the tool employs TIP3P water model and AMBER99SB-ILDN forcefield [16], though the forcefields can be changed by a user. If checkpoint files *${protein-name}.gro* and *topol.top* already exist in the working directory (*root/md_files/md_preparation/protein/*) the preparation step will be skipped.

### Force field selection

In StreaMD, users can specify a force field from the GROMACS short names list by providing the *–protein_forcefield* argument (e.g., *amber99sb-ildn*). Any of the default GROMACS force fields can be selected, as they are stored in the *forcefield.itp* files located within the < *forcefield* >*.ff* subdirectories of the GROMACS Conda library directory (e.g., *Miniconda3/envs/md/share/gromacs/top*). For the use of non-standard force fields, users can place custom < *forcefield* >*.ff* subdirectories, containing the necessary *forcefield.itp* files, either in the GROMACS Conda library directory or in the current working directory. The working directory can be specified using the *--wdir* argument.

### Ligand/cofactor preparation

If a user supplies 3D structures of ligands or cofactors, the tool initiates a molecular preparation step, generating *mol2* files containing coordinates and atomic charges, along with corresponding *${ligand-id}.itp* files encompassing force field constants and *posre_${ligand-id}.itp* files specifying restraints for equilibration. Molecule preparation starts with addition of hydrogens according to the charged states of atoms and the total formal charge. For molecules incorporating boron atoms lacking force field parameters, a special workflow for geometry optimization and electrostatic potential computation was implemented, utilizing Gaussian software (http://signe.teokem.lu.se/ulf/Methods/resp.html, https://www.x-mol.com/groups/Dong/news/816). Gaussian output files are transformed into *mol2* format with calculated RESP charges by the antechamber tool. To employ the Gaussian parameterization approach, users are required to submit the path to the Gaussian executable file and an activation string for the Gaussian module (if computations are to be conducted on a cluster). For other molecules, antechamber is utilized to compute bcc charges and generate *mol2* files.

The generated *mol2* files serve as input for Amber parmchk2, facilitating the creation of force field modification files (*frcmod*) containing requisite force field parameters. Subsequently, the LEaP program (*tleap*) is employed to generate AMBER topology and coordinate files, which are subsequently converted into GROMACS topology and coordinate files using ParmED. Finally, *gmx genrestr* is utilized to generate position restraints for each prepared molecule.

Any encountered issues during the preparation of individual ligands will not impact others, as unprepared ligands are simply omitted from the process. Conversely, any complication arising with a cofactor will halt program execution, as a system cannot undergo simulation without all cofactors present.

The presence of *${ligand-id}.itp* and *posre_${ligand-id}.itp* files in the corresponding directory will trigger the bypassing of the molecule preparation step. If *mol2* files exist without accompanying *itp* files, the preparation workflow exclusively skips the *mol2* generation step, including the Gaussian-based process for molecules containing boron atoms. This may also work for molecules containing other atoms, but we did not investigate this possibility.

### Complex preparation

Following the prior steps, all prepared files including those for the protein, ligands, and cofactors are seamlessly merged into corresponding *complex.gro* and topology files, which are then stored within a designated *md_run* directory. The solvation process is executed by *gmx solvate*, configuring a cubic box with a 1 nm distance between the solute and the box. To neutralize the system, Na + and Cl- ions are introduced via *gmx genion*. A checkpoint file, *solv_ions.gro*, is generated accordingly. In cases where this file exists, both the solvation and neutralization steps are automatically skipped.

For protein–ligand complexes involving metal ions, a distinct preparation protocol utilizing the MCPB.py module [17] was implemented. Application of the MCPB.py parametrization necessitates user provision of metal residue names, alongside specification of the Gaussian executable file path and the Gaussian module activation string (particularly for cluster-based computations). It should be noted that the behavior of MCPB.py is highly system-dependent. Users are advised to have a thorough understanding of the procedure before using it, and the procedure should be applied with caution.

System minimization proceeds until the maximum force value reaches 1000.0 kJ/mol/nm or less, but not exceeding 50,000 steps. Following this, consecutive 1000 ps NVT and NPT equilibrations are executed (the time duration can be customized by a user), and position restraints are automatically applied to the heavy atoms of both protein, ligand and cofactors. Minimization and equilibration phases yield respective system analysis files, such as p*otential.png* and *potential.csv* detailing potential energy variations during minimization, and *temperature.png/csv*, *pressure.png/csv*, and *density.png/csv* from the equilibration phase. These files serve to visually assess system stability and facilitate further analysis. Throughout these procedures, the tool generates checkpoint files to expedite subsequent runs by skipping completed minimization, NVT, or NPT equilibration steps.

### MD simulations

Users have the option to define the simulation duration in nanoseconds, with a default value of 1 ns, as this is a minimum reasonable trajectory length to perform some analysis and identify issues. The outcome of this phase comprises *md_out.tpr* (topology), *md_out.xtc* (trajectory), and *md_out.cpt* (checkpoint) files. If these files exist the system processing will be automatically continued until the required time of simulation will be reached, this procedure would be accompanied with the corresponding warning message. To resume an interrupted simulation or extend a completed one, users can specify the path (or paths) to the directory containing *xtc*, *tpr*, and *cpt* files from previous simulations. Additionally, they can supply a new simulation duration in nanoseconds.

### Customized molecular dynamics parameter (.mdp) files

While StreaMD is designed with default, suboptimal parameters to accommodate a wide range of system simulations, certain cases may require more specific configurations. For such instances, StreaMD allows users to customize parameters for neutralization (ion addition), energy minimization, NVT, NPT, and production simulation steps. To utilize custom parameters, users can provide the *--mdp_dir* argument, specifying the directory that contains one or more Molecular Dynamics Parameter (.mdp) files. These files should retain their system-specific names, which can be found in the *streamd/scripts/mdp* directory. If any .mdp files are not provided, the missing files will automatically be sourced from the default parameter directory.

### Replicas

Repeating the simulation multiple times allows for better statistical sampling of the space, providing more reliable averages and insights into the system's behavior. By default, StreaMD does not support multiple repetition within the same run. Although a user can perform multiple separate runs by applying the same command with different working directory argument (*--wdir*) and if needed different setup seed.

### MD analysis

In this phase, the tool undertakes system centering, alignment, and elimination of periodic boundary conditions to yield a trajectory amenable for subsequent MM-GBSA/PBSA calculations and for the retrieval of protein–ligand fingerprints. Consequently, the tool generates a *frame.pdb* file containing the tenth frame of the trajectory for the entire system, alongside *md_short_forcheck.xtc*, which constitutes a subset of the complete trajectory (every 50th frame if the trajectory length is 10 ns or less, and every 100th frame if the trajectory is longer). These files serve for fast visual inspection of the obtained trajectory. Furthermore, the tool calculates root-mean-square fluctuation (RMSF) and radius of gyration for the protein, and root-mean-square deviation (RMSD) values for the protein backbone, active site residues within 5 Å of the ligand and the ligand itself, individually assessing each cofactor as well. The computed data is saved in CSV and PNG format (by seaborn module), facilitating the subsequent analysis.

### Trajectory convergence analysis

We implemented a dedicated module, *run_analysis*, to calculate trajectory parameters that can assist in identifying converged segments of molecular dynamics trajectories. This data is valuable for subsequent MM-GBSA/PBSA analyses. Specifically, we suggest calculating the average root-mean-square deviation (RMSD) of the ligand, protein, and active site residues within 5 Å of the ligand, as well as the standard deviation of RMSD for the same trajectory segment. The average RMSD provides insight into ligand movement or rotation relative to its initial pose, while the standard deviation reflects the stability of the ligand pose within the selected trajectory segment.

Users can specify the desired trajectory segment in nanoseconds, and the analysis will generate a *csv* output file containing this data, alongside an *html* file with interactive visualizations. For MM-GBSA analysis, it is reasonable to focus on the final segment of the trajectory to assess ligand pose stability. Users have the flexibility to select the trajectory segment for analysis. The same parameters (average RMSD and its standard deviation) are also automatically computed for the protein and binding site residues within 5 Å of the ligand. This information can further be utilized to exclude complexes where the protein displays unstable behavior.

### MM-GBSA/PBSA calculation

The *run_gbsa* module offers a straightforward interface for computing binding free energy using the gmx_MMPBSA tool [18]. To start calculations a user should supply the directories containing simulation outputs generated by StreaMD or external trajectory (*xtc*), topology (*tpr*) and *index.ndx* files. Users have the option to either customize a file containing parameters for MM-GBSA/PBSA calculations (*mmpbsa.in*) or supply their own input file. Upon completion of calculations for all ligands, the module automatically parses and merges outputs in a unified aggregated output file. To facilitate efficient parallel processing, *run_gbsa* utilizes Dask library, dynamically determining the number of processes allocated for each calculation based on the number of frames utilized in the trajectory.

The accuracy of binding free energy calculations depends on multiple factors. The most important are continuum solvation model, interior dielectric constant or entropy treatment. In the present study Interaction Entropy (IE) was used to approximate the binding entropy. IE is computationally very efficient and relatively accurate approach [19]. However, accuracy of entropy estimation can vary substantially for complex and highly flexible systems [20], therefore, some authors prefer to not perform entropy calculation at all [21]. To specify another approach for entropy calculation (quasi-harmonic entropy (QH) or a normal mode analysis) a user may supply a custom *mmpbsa.in* file.

Meanwhile the correct value of interior dielectric constant may also have significant impact on the estimation of solvation energy especially for simulations of polar or charged molecules. The solute interior dielectric constant value equals 1 is usually used by default, although some works show that it can result in an overestimation of the ligand–receptor electrostatic interaction for some systems and values 2–4 often perform better especially in large data sets of diverse proteins or charged systems [6]. In our pipeline we set up the value of interior dielectric constant to 4 by default, although a user should take into account that the best dielectric constant is system-dependent and some parameter scanning may be required to achieve the highest accuracy.

### Protein–ligand fingerprint analysis

The *run_prolif* module facilitates the extraction of protein–ligand contacts through utilization of the ProLIF python library [22]. To start the analysis, users are required to supply directories containing simulation outputs generated by StreaMD or external trajectory (*xtc*) and topology (*tpr*) files. Leveraging Dask for parallel processing, the module enhances computational efficiency. The primary output consists of a text file (*plif.csv*) within each simulation directory, documenting all identified contacts for each trajectory frame. This default behaviour can be customized by adjusting the step parameter to select every n-th frame for analysis. Subsequently, the extracted data is visualized in a 2D plot (*plif.png*) by plotnine module. Additionally, an interactive 2D interaction network (*plif.html*) is generated, showing detected protein–ligand contacts. By default, all contacts will be visualized. This may be misleading in cases if a ligand moves a lot and some contacts cannot be actually established simultaneously. However, users have the flexibility to modify the minimum frequency of occurrence of displayed contacts. Further, protein–ligand interaction fingerprints for all complexes are consolidated into a single file (*prolif_output.csv*), along with a 2D plot (*prolif_output_occupancy0.6.png*) illustrating protein–ligand contacts with a specified minimum occupancy. Users can adjust the default occupancy threshold of 0.6 to suit their preferences.

### Logging of calculations

To facilitating identification of issues and tracking the progress StreaMD provides two levels of logging. The general information about each step (e.g. the passed arguments, running and finished steps) is collected in a single log-file placed in the *root* directory of the project. The outputs of individual programs (e.g. GROMACS, Antechamber, Gaussian) are collected in separate log-files individual for every processing system and they are located in the corresponding directories of simulating systems. Additional tools (MM-GBSA/PBSA and ProLIF) also produce log-files: one in the *root* directory of the project (or a directory from where the script was launched) and separate log-files for individual systems which are stored in the corresponding directories. Therefore, if there are any errors reported in the general log-file, a user may look at particular log-files to identify an issue.

### GPU acceleration

The StreaMD tool supports energy minimization, NVT, and NPT equilibration steps, as well as production simulations on GPU(s), which can significantly improve computational speed and efficiency. By using the *--device gpu* argument, users can offload all eligible computations (including nonbonded interactions, updates, PME, bonded forces, and PMEFFT) to GPU. Alternatively, users can enable GROMACS to automatically optimize the distribution of calculations between the CPU and GPU by setting the *--device auto* argument, which may be advantageous on systems where the CPUs usage offers comparable performance to GPUs. GPU-based calculations are also compatible with distributed environments across multiple servers equipped with GPUs, without requiring any additional user actions.

## Results and discussion

The wide functionality of the tool makes it useful for different practical tasks. The tool has been successfully applied in a number of in-house studies, however only few of them have been published so far [23–25]. Below we will demonstrate the utility of StreaMD on cofactor-containing systems, several benchmark datasets and study the computational performance.

### Cofactor-containing systems

In our previous studies [24, 25], we explored estradiol dimers, a class of compounds with anticancer properties that act by binding to the colchicine-binding site, thereby inhibiting tubulin polymerization and disrupting mitotic spindle formation. Molecular docking was conducted using the tubulin-colchicine complex (PDB ID: 4O2B), retaining key cofactors such as GTP and Mg^2+^ ions, which are critical for the regulation of polymerization. To further validate the stability of the docking poses and identify the key interactions responsible for the observed biological activity, we performed 150 ns molecular dynamics (MD) simulations and calculated the binding free energies. The computed free energies were consistent with experimentally measured tubulin polymerization rates, yielding a correlation coefficient of 0.93. These findings corroborated the validity of the predicted binding poses and the established protein–ligand interactions.

### GBSA energy calculation

To assess the functionality of the implemented tool, we conducted 10 ns single run simulations and computed Generalized Born Surface Area (GBSA) energies for complexes sourced from the Greenidge dataset [14]. Molecules underwent automatic preparation and pre-processing using the default StreaMD protocol with GPU support. We successfully executed simulations for 624 out of 626 complexes. 1SRG prepared complex accidently consisted of only 3 amino acids and 1NJE complex returned a convergence error during the ligand preparation step from antechamber, which we have not succeeded to solve.

**Fig. 2:**
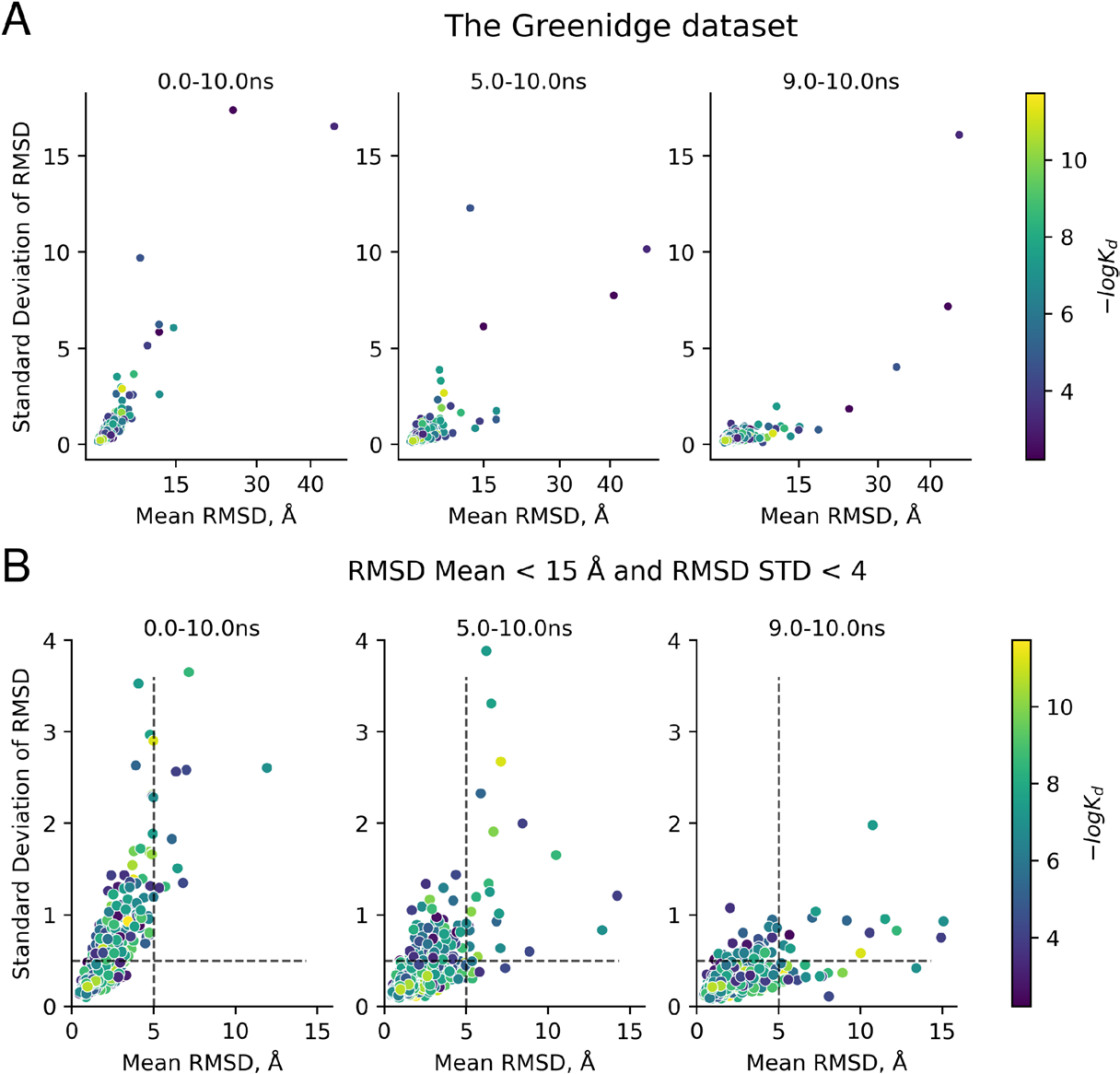
Distribution of average RMSD values for a ligand and standard deviation of RMSD across different segments of trajectories: 0–10 ns, 5–10 ns, 9–10 ns. The top figure depicts all data points (**A**), the bottom one is its zoom (**B**). Dashed lines designate the proposed thresholds for selection of complexes with converged ligand trajectories

Prior to conducting MM-GBSA calculations, we assessed the convergence of ligand trajectories across various time frames. Specifically, we analyzed the entire trajectory, as well as the last 5 ns and 1 ns segments. Ligands that dissociated from the binding site exhibited high average RMSD values and large RMSD standard deviations, indicating significant changes in their position or orientation within the selected trajectory segments. Such ligands typically displayed low to moderate binding affinities (Fig. 2). A threshold of 5 Å was chosen as a potential cutoff for the average RMSD to identify dissociating ligands. Additionally, to exclude ligands that remained in the binding site but exhibited excessive movement, we proposed using a 0.5 Å threshold for the RMSD standard deviation. This criterion ensures that RMSD fluctuations remain within 2 Å for 95% of the selected trajectory (corresponding to 2σ).

**Fig. 3:**
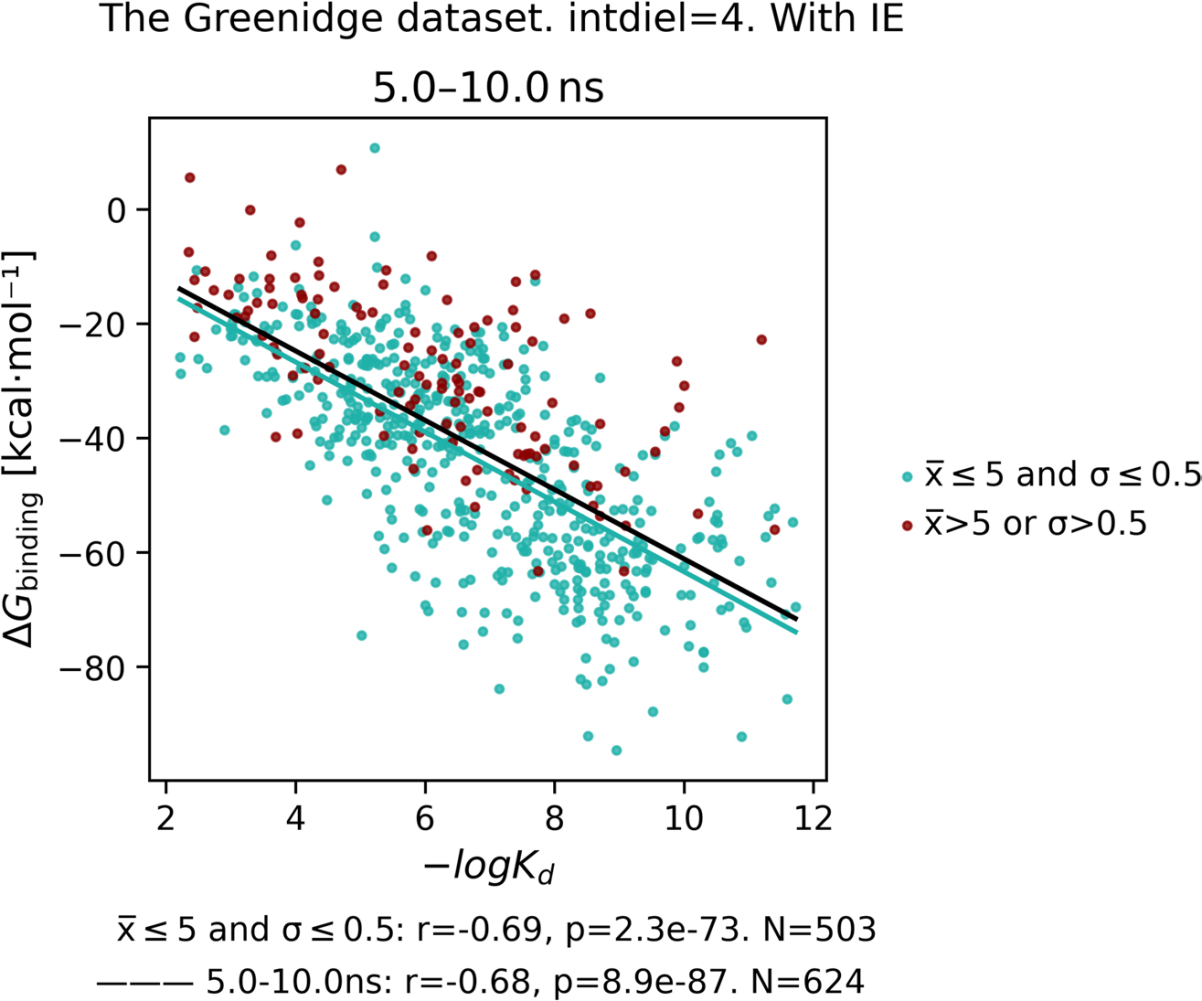
Correlation between calculated MM-GBSA free energies and observed pK_d_ (Pearson R = −0.69) for 624 protein–ligand complexes from the Greenidge data set [14]. Free energies were calculated from the last 5 ns of the trajectories using each fifth frame and internal dielectric constant 4. Red dots are compounds which have not converged trajectories: average RMSD > 5 Å or standard deviation of RMSD > 0.5 Å

For the calculation of binding free energies, we suggested selecting the final 5 ns of the trajectory (i.e., 5 to 10 ns), as this longer segment is expected to provide more reliable and robust results (Fig. 3). In this particular case, however, the output correlations showed little sensitivity to the selected trajectory segment (Table S1), likely because all ligands were simulated from their native poses (Figure S1). Nonetheless, in practical applications where the true ligand poses are unknown, this type of analysis is crucial for determining appropriate thresholds and selecting the most suitable trajectory segment. Additionally, omitting the interaction entropy (IE) term, as demonstrated in the reference study by Gomes et al. [14], had a negligible effect on the correlation results (Figure S1).

To further validate the default protocol, we selected three curated datasets of high-quality PDB complexes sourced from the work of Bahia et al. [26]. These datasets encompassed 166 protein–ligand complexes for human β-secretase 1 (UniProt ID: P56817), 63 complexes for human α-thrombin (UniProt ID: P00734), and 51 complexes for bovine trypsin (UniProt ID: P00760). Within each dataset, we identified a reference complex characterized by minimal root-mean-square deviation (RMSD) to all other complexes and high resolution (β-secretase 1: PDB 3UFL, α-thrombin: PDB 4AYY, and bovine trypsin: PDB 1O2I). Subsequently, all other complexes were aligned to their respective reference structures to obtain initial ligand coordinates. Clashes of ligands after alignment were automatically solved during the equilibration and minimization steps, thus no explicit intervention was required. Subsequently, we conducted 10 ns molecular dynamics (MD) simulations for each complex. To compute GBSA binding free energies, we varied dielectric constants (intdiel = 1 or 4) and considered or disregarded the interaction entropy term.

**Fig. 4:**
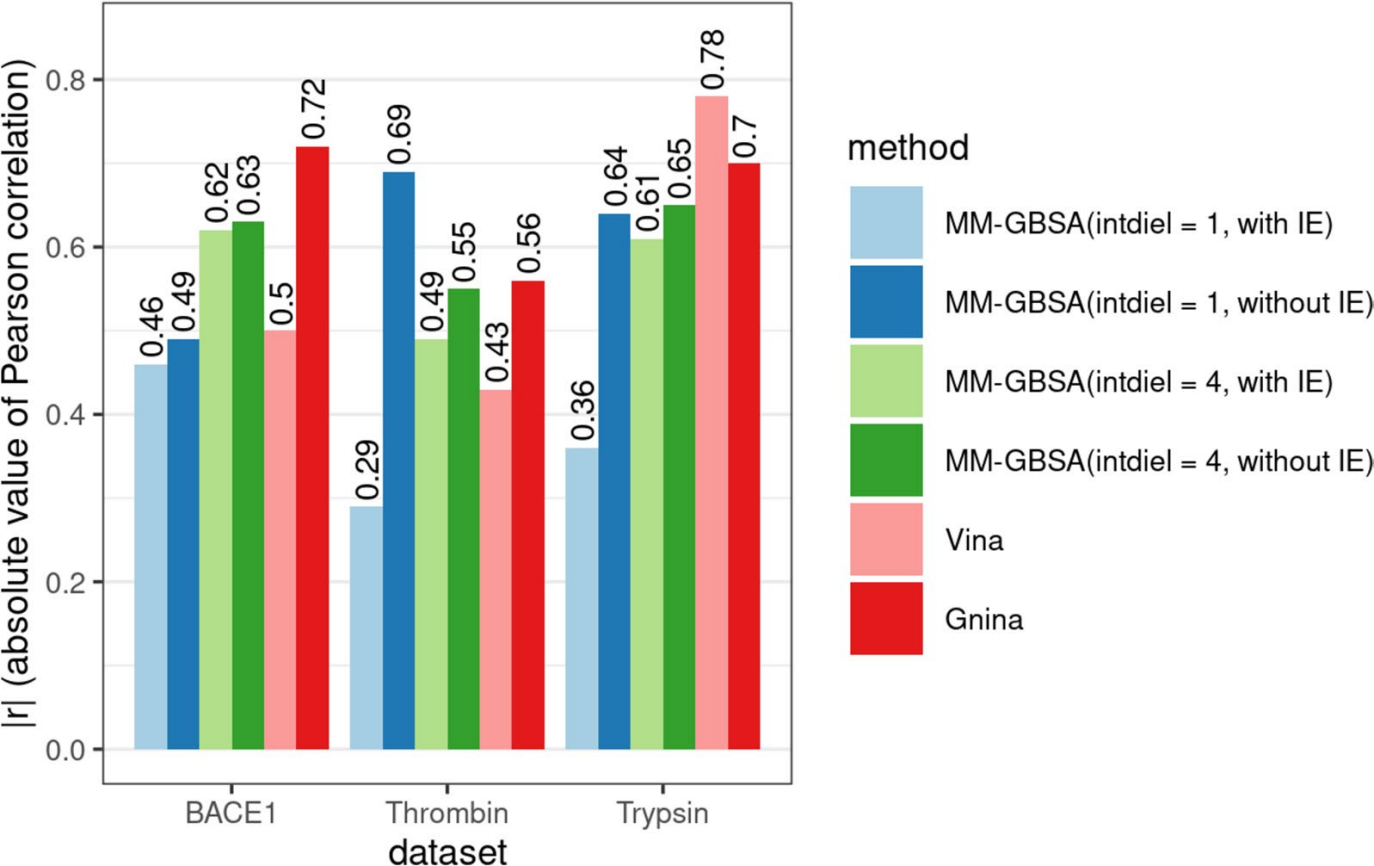
Correlation between docking scores or calculated MM-GBSA free energies with experimental affinities for three benchmark data sets. MM-GBSA free binding energies were calculated for different dielectric constants (1 or 4) and considering or ignoring the interaction entropy term (with or without IE). Only compounds satisfying chosen criteria (average RMSD ≤ 5 Å and standard deviation of RMSD ≤ 0.5 Å) were used to calculate correlations. Scatterplots between docking scores or calculated free energies and experimental pK_d_ values are available in Figures S2-S7. Correlation statistics for other trajectory segments and for all compounds is in Figure S9

For the calculation of binding free energies, we utilized the final 5 ns of the trajectories, focusing exclusively on compounds that met the previously established criteria: average RMSD ≤ 5 Å and standard deviation of RMSD ≤ 0.5 Å. The number of compounds that satisfied these criteria was 134 for β-secretase 1, 58 for thrombin, and 32 for trypsin. Based on our analysis (Fig. 4), excluding the entropy term proved beneficial in all cases. Furthermore, when the dielectric constant was set to 1 (intdiel = 1), omitting the entropy term substantially improved the correlation. This improvement may be attributed to larger errors in the calculated binding free energy when the entropy term is included (Figures S10 and S11). These results indicate that the entropy term introduces significant errors at a dielectric parameter of 1, whereas no such dependency is observed at intdiel = 4. Consequently, these findings suggest that the inclusion of the interaction entropy term may not be essential for effectively ranking a large set of compounds, as it does not substantially enhance the ranking process.

For comparative purposes, we conducted molecular docking utilizing Vina [27] and Gnina [28] (dense_ensemble model) integrated in EasyDock [15]. In both cases, the exclusiveness parameter was set to 32. Notably, for the trypsin dataset, both docking programs surpassed the MM-GBSA approach in their ability to rank compounds. In the case of the β-secretase dataset, docking with Gnina exhibited comparable performance to MM-GBSA, while Vina demonstrated inferior performance. Conversely, for the thrombin dataset, a particular setup of MM-GBSA (intdiel = 1 and without interaction entropy) yielded superior ranking capability, followed by Gnina and Vina. While Vina and Gnina demonstrated commendable performance, it's worth noting that this might be attributed to the inclusion of these compounds in the training of these models. Notably, Vina achieved the highest performance on the trypsin dataset, with a significant proportion of these complexes published well before the release of Vina (Figure S8). A similar situation applies to Gnina, as all of these complexes are part of the PDBbind refined set v2019, which was utilized for training the convolutional models of Gnina. Thus, despite its higher computational demands, the MM-GBSA approach may offer advantages in certain scenarios, outperforming state-of-the-art docking tools. However, it may necessitate parameter tuning for optimal performance.

### Scalability and general performance

To assess the scalability of StreaMD, we conducted 51 simulations of the Trypsin dataset, with each simulation comprising 1 ns for NVT and NPT equilibration steps, followed by an additional 1 ns for the production simulation. These simulations were executed in both single-node and multiple-node modes, utilizing a total of 13 nodes, each equipped with 128 CPU cores.

In the single-node mode, the entire process, including preparation, 1 ns MD simulation, and analysis, required 1026 min for the 51 complexes. In contrast, the multiple-node mode completed the same tasks in 90 min. The calculated overhead was 14%, primarily attributed to the fact that during the preparation and analysis stages, a single molecule is processed on a single CPU core. Given that there were only 51 ligands, not all nodes were fully occupied during these stages, resulting in the observed overhead. However, the simulation stage demonstrated perfect parallelization, efficiently utilizing all cores on all nodes as expected.

To address this issue, we introduced a specific argument to the program interface, allowing users to selectively execute one of three stages (preparation, simulation, analysis). This flexibility enables users to conduct the preparation step separately on a single server, while simulations can be concurrently executed on all servers in a separate run. By default, all steps are sequentially executed, commencing from input structures of proteins and ligands and concluding with the analysis of obtained trajectories.

To evaluate the performance enhancement achieved through GPU acceleration, we conducted the same calculations on a single node equipped with one GPU (NVIDIA A100-SXM4-40 GB) and 12 CPUs, where all possible computations (including nonbonded interactions, updates, PME, bonded forces, and PMEFFT) for minimization, NVT, NPT, and production runs were offloaded to the GPU. The total calculation time for 51 complexes was 514 min, reflecting approximately a 200% speedup compared to a node with 128 CPUs.

### Analysis of protein–ligand interactions

**Fig. 5:**
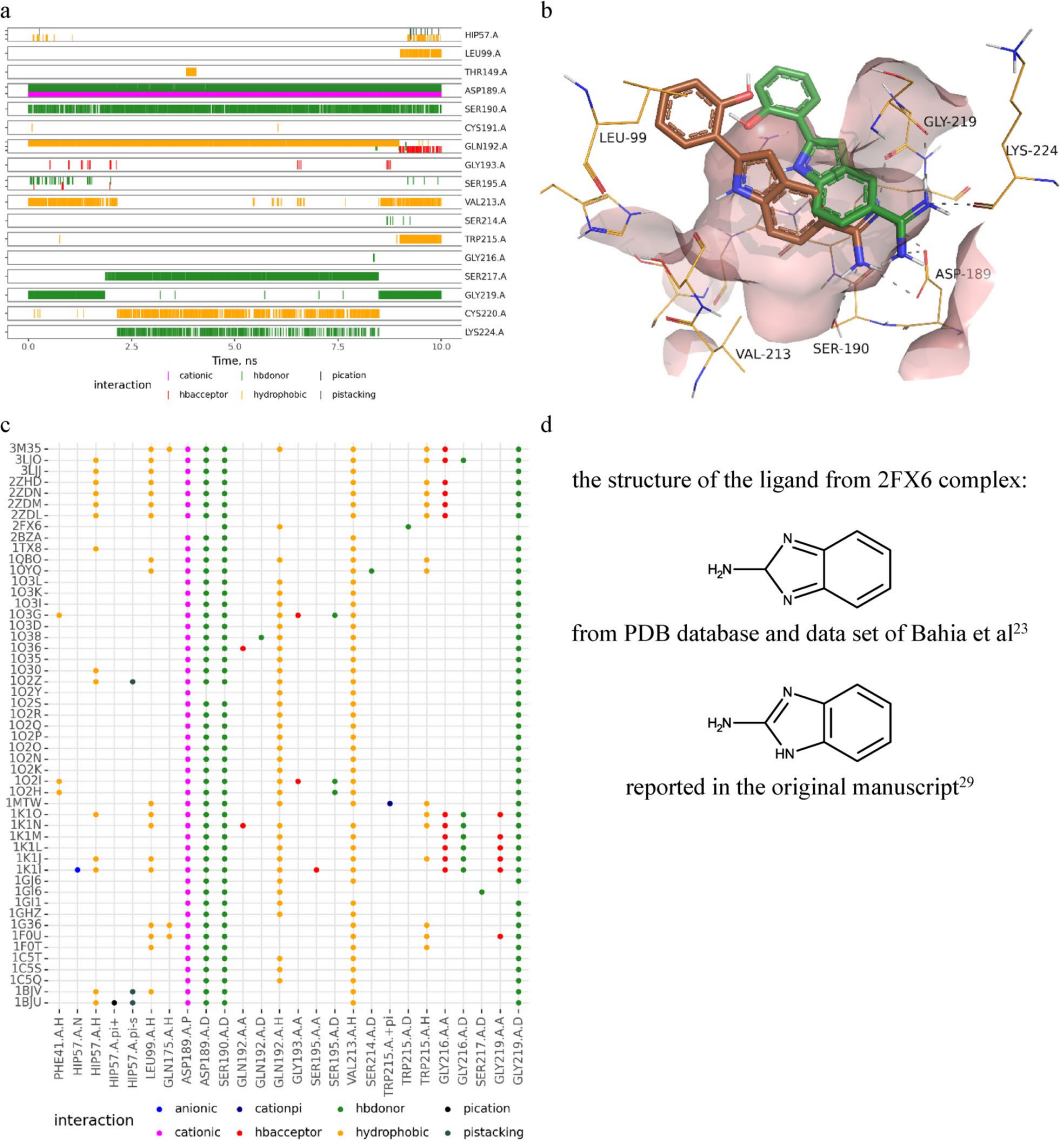
Protein–ligand interactions detected for the trypsin dataset. **a** Interaction fingerprints detected for 1GI6 protein–ligand complex during 10 ns MD simulation. **b** Starting and finishing poses (orange) and the pose in the middle of the simulation (green) of 1GI6 protein–ligand complex during 10 ns MD simulation. **c** Interaction fingerprints for the whole trypsin dataset occurred in at least 60% of frames of 10 ns MD trajectories. **d** Structures of the ligand from 2FX6 complex annotated in PDB and the dataset of Bahia et al. [23] and in the original manuscript [29]

An additional analysis of protein–ligand contacts can be performed using ProLIF. The outputs can be visualized for individual protein–ligand systems as well as for a set of systems. We demonstrated these outputs for the dataset of trypsin inhibitors. The analysis of individual protein–ligand systems may show which contacts are co-occurred and how these groups of contacts change during the simulation that may suggest ligand moving or pose changing. There is an example of the analysis of an individual trajectory in Fig. 5a. The ligand 1GI6 complex forms typical strong interactions with Asp189 and Ser190 of trypsin. Additionally, there are an H-bond with Gly219 and a hydrophobic interaction with Val213. These additional contacts are broken after 2 ns and new contacts are established with Ser217, Cys220 and Lys224. However, after 8 ns the ligand again creates contacts with Val213 and Gly219 along with new ones (Leu99, Trp215). These changes in contacts indicate changes in ligand poses. The ligand after starting of the simulation goes deeper into the binding site and afterwards returns back to the initial pose (Fig. 5b).

The analysis of contacts observed for multiple ligands may help to identify the most frequently observed contacts and interaction patterns and identify ligands which do not follow them, that may indicate their unique binding modes or issues in a simulation setup. The analysis of the whole set of trypsin inhibitors revealed as expected the common interaction pattern. The majority of ligands have charged interaction with Asp189, H-bonds with Ser190 and Gly219 and hydrophobic interactions with Gln192 and Val213 (Fig. 5c). However, a ligand from 2FX6 complex did not follow this pattern. Visual inspection of a ligand MD trajectory revealed that the structure of the ligand was wrongly annotated in the PDB database and was not fixed in the dataset collected by Bahia et al. (Fig. 5d). The bond orders were incorrectly interpreted, that results in wrong geometry of the structure and that the ligand started to move away from its initial pose and could not form expected contacts. These simple examples demonstrate how the analysis of protein–ligand interactions may be used to retrieve important and useful information about simulated systems.

### StreaMD options and features:

- Default set of optimal parameters to run molecular dynamics, which can be customized
- Support of simulations of different molecular systems in explicit water solvent:
  - Protein
  - Protein-cofactor(s)
  - ⦁protein-ligand
  - Protein–ligand-cofactor(s)
- Support of modeling of boron-containing molecules (using the Gaussian program)
- MCPB.py support to simulate proteins with specific metal ions not parametrized in commonly used force fields
- The ability to continue interrupted simulations or to extend finished ones
- Support of distributed computing using Dask library across a network of severs (not necessary a cluster)
- Automatic analysis of simulations:
  - RMSD plot for protein, active site, ligand and cofactors objects
  - A plot of flexibility of side chains of amino acids (RMSF)
  - A plot and a pdb file with radius of gyration
  - A single frame pdb file for the topology and a short subset of the trajectory for the quick visual inspection
  - A fitted trajectory (with removed periodic boundary conditions, aligned and centered on the first frame) to use for energy or protein–ligand interaction calculations
- Support of analysis of MD trajectories by additional instruments:
  - ProLIF: Ligand–Protein interactions
  - MM(PB)GBSA: Calculation of Binding Energy
  - Logging of every calculation running

### StreaMD limitations and remarks:

- Preparation of boron-containing molecules and the MCPB.py protocol requires a Gaussian license;
- Running a protocol on the number of molecules less than the total number of cores on multiple servers can be inefficient due to inability to distribute the antechamber ligand preparation tasks among more than 1 computational core per ligand;
- StreaMD , as well as a conda version of GROMACS, can be run only on Linux.

## Conclusions

We have implemented a comprehensive automated pipeline capable of conducting molecular dynamics (MD) simulations utilizing GROMACS, calculating binding free energies employing the MM-GBSA/PBSA methodology, and generating protein–ligand interaction fingerprints using ProLIF. The main feature of the developed tool is that it does not require deep knowledge of molecular dynamics and GROMACS. The tool accommodates simulations involving proteins, protein–ligand complexes, and cofactors, with seamless handling of complexes containing specific metal ions (via MCPB.py) and boron-containing ligands (via Gaussian). Furthermore, computations can be efficiently distributed across servers within a network or cluster, facilitated by the Dask Python library with minimal overhead.

Through testing on number of benchmark datasets to evaluate binding free energies using the Generalized Born Surface Area (GBSA) method, we have identified default parameters: employing a dielectric constant of 4 and disregarding the entropy term. The exclusion of the entropy term was recommended due to its marginal impact on enhancing ranking performance, while imposing a computational burden.

Our developed tool holds versatile applicability across diverse scenarios, with particular potential for performing large-scale simulations, such as the calculation of binding free energies utilizing the MM-GBSA/PBSA approach for a substantial number of ligands.

## Supplementary Information

Supplementary material 1.

Supplementary material 2.

## Data Availability

Data is provided within the manuscript or supplementary information files.

## Availability and requirements

Project name: StreaMD

GitHub: https://github.com/ci-lab-cz/streamd

Operating system(s): Linux

Programming language: Python 3

Other requirements: GROMACS, RDKit, ProLIF, Antechamber, MDAnalysis, Dask, Gaussian (optional, a license is required)

License: MIT

Any restrictions to use by non-academics: no
