# Supplementary material for: StreaMD: the toolkit for high-throughput molecular dynamics simulations
Source: J Cheminform. 2024 Nov 5;16:123. doi: 10.1186/s13321-024-00918-w (PMC11539841; doi:10.1186/s13321-024-00918-w)
Supplement: Supplementary file 2 — Supplementary material 2. [file 13321_2024_918_MOESM2_ESM.pdf]

Supplementary Information

Tables S1. Pearson Correlation table. The Greenidge dataset. 624 complexes. Molecular Mechanics/Generalized Born Surface Area (MM/GBSA). The MM/GBSA internal dielectric constant (*intdiel*) was set to 4.0 (the same as in the reference paper https://pubs.acs.org/doi/10.1021/acs.jcim.2c00919). Pearson correlation coefficient was calculated for each method separately for two groups of compounds, one where $\bar{x}$ (the average root-mean-square deviation (RMSD) of the ligand) is less or equals 5Å as well as σ (standard deviation of RMSD of the ligand) is less or equals 0.5Å and another with all compounds for different trajectory segments.

	Start, ns	End, ns	Step	Number of frames	Number of molecules		Pearson's r (with IE)		Pearson's r (without IE)		Pearson's r (ChemFlow*, 18 ns)	
Compounds					$\bar{x}\leq5$ and $\sigma\leq 0.5$	All	$\bar{x}\leq5$ and $\sigma\leq 0.5$	All	$\bar{x}\leq5$ and $\sigma\leq 0.5$	All	$\bar{x}\leq5$ and $\sigma\leq 0.5$	All
StreaMD	0	10	5	201	422	624	-0.69	-0.68	-0.69	-0.71	-0.69	-
	5	10	5	101	503		-0.69	-0.68	-0.69	-0.69	-0.7	-
	9	10	1	101	556		-0.69	-0.68	-0.68	-0.68	-0.7	-
ChemFlow*	0	18*	-	-	-		-	-	-	-	-	-0.72

* Correlation was calculated based on data from https://pubs.acs.org/doi/10.1021/acs.jcim.2c00919

Table S2. Pearson correlations between calculated docking scores or MM-GBSA free energies for 10 ns trajectories and experimental pK_d values. Pearson correlation coefficient was calculated for each method separately for two groups of compounds, one where $\bar{x}$ (the average root-mean-square deviation (RMSD) of the ligand) is less or equals 5Å as well as σ (standard deviation of RMSD of the ligand) is less or equals 0.5Å and another with all compounds for different trajectory segments.

Dataset*	Start, ns	End, ns	Step	Number of frames	intdiel	Number of molecules		Pearson's r (with IE)		Pearson's r (without IE)		Gnina		Vina	
Compounds						$\bar{x}$≤5 and σ≤ 0.5	All	$\bar{x}$≤5 and σ≤ 0.5	All	$\bar{x}$≤5 and σ≤ 0.5	All	$\bar{x}$≤5 and σ≤ 0.5	All	$\bar{x}$≤5 and σ≤ 0.5	All
BACE1	0	10	5	201	1	86	166	-0.38	-0.5	-0.47	-0.49	0.62	0.72	-0.41	-0.58
					4			-0.55	-0.65	-0.59	-0.66				
	5	10	5	101	1	134		-0.46	-0.48	-0.49	-0.48	0.72		-0.5	
					4			-0.62	-0.64	-0.63	-0.65				
	9	10	1	101	1	146		-0.47	-0.47	-0.46	-0.46	0.71		-0.48	
					4			-0.6	-0.63	-0.61	-0.64				
Thrombin	0	10	5	201	1	54	63	-0.42	-0.46	-0.73	-0.69	0.56	0.58	-0.46	-0.43
					4			-0.51	-0.56	-0.55	-0.6				
	5	10	5	101	1	58		-0.29	-0.3	-0.69	-0.68	0.56		-0.43	
					4			-0.49	-0.53	-0.55	-0.59				
	9	10	1	101	1	61		-0.45	-0.46	-0.64	-0.67	0.56		-0.42	
					4			-0.5	-0.54	-0.54	-0.58				
Trypsin	0	10	5	201	1	26	51	-0.49	-0.41	-0.69	-0.57	0.74	0.65	-0.81	-0.73
					4			-0.67	-0.57	-0.7	-0.6				
	5	10	5	101	1	32		-0.36	-0.28	-0.64	-0.59	0.7		-0.78	
					4			-0.61	-0.56	-0.65	-0.63				
	9	10	1	101	1	41		-0.5	-0.46	-0.59	-0.59	0.69		-0.78	
					4			-0.62	-0.62	-0.63	-0.63				

* Bahia, M. S., et. al. (2023). A comparison between 2D and 3D descriptors in QSAR modeling based on bio-active conformations. Mol. Inf., 42(4), 2200186.

Table S3. StreaMD default values of parameters.

Step	Parameter	Default value
Minimization	Stop minimization when the maximum force < (emtol)	1000.0 kJ/mol/nm
NVT/NPT equilibration	temperature for Maxwell distribution	310 K
	Position restraints	Heavy atoms of protein and ligand
MD simulation	reference temperature	310 K
gmx_MMPBSA	startframe	1
	endtframe	100 (1 ns)
	interval	1
	ie_segment	100%
	interaction_entropy	1 (use by defualt)
	temperature	310 K
	PBRadii	3
gmx_MMPBSA (GB calculation)	igb	5
	saltcon	0.15
	intdiel	4
ProLIF	Fingerprints	Hydrophobic, HBDonor, HBAcceptor, Anionic, Cationic, CationPi, PiCation, PiStacking, MetalAcceptor

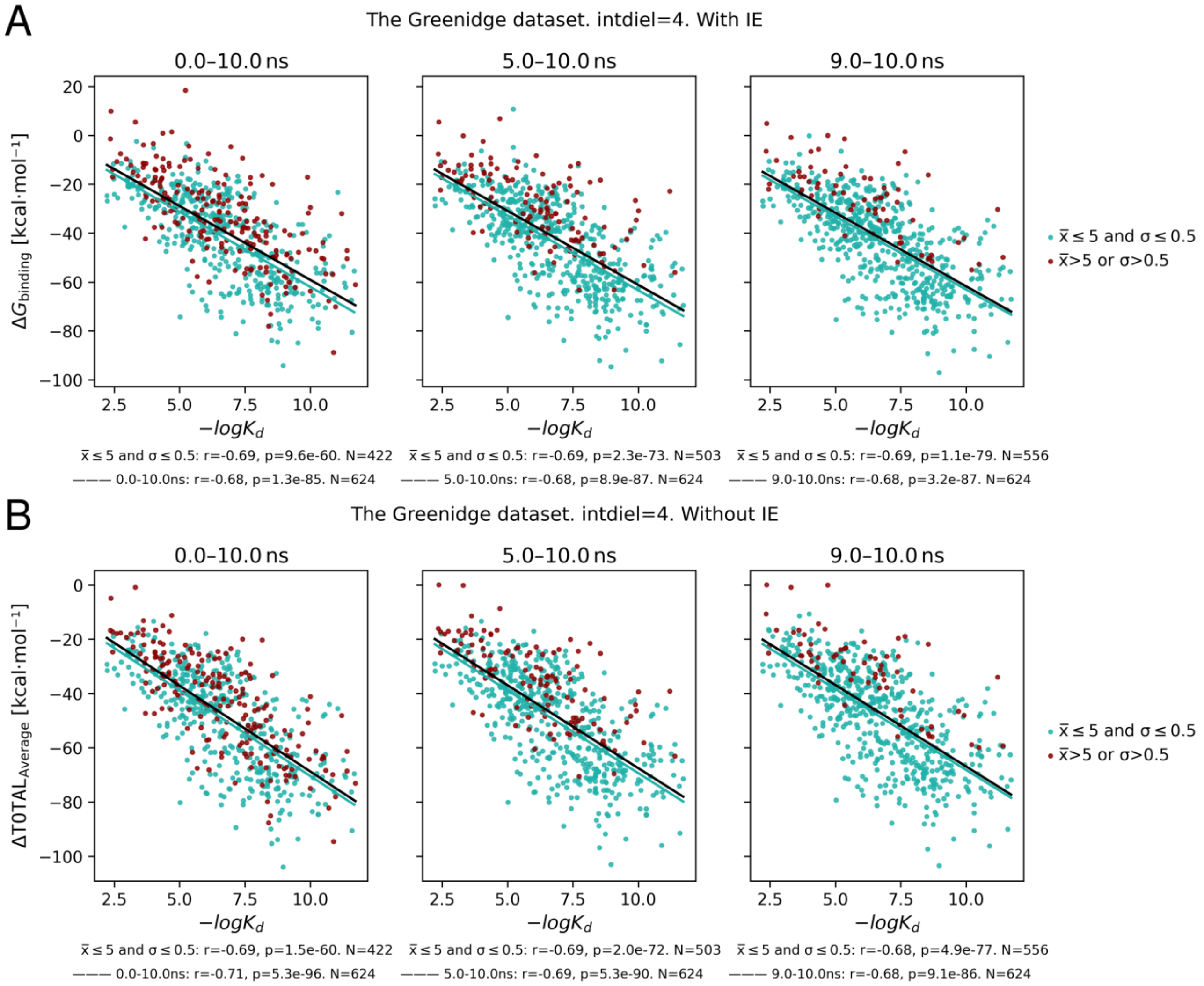

Figure S1. Correlation between experimental affinity values (-logK_d) of Greenidge dataset ligands and the calculated GBSA binding free energies taking into account interaction entropy (IE) term (**A**) and not (**B**) for different trajectory segments. The MM/GBSA internal dielectric constant (*intdiel*) was set to 4.0. Pearson correlation coefficient was calculated for each method separately for two groups of compounds, one where x̄ (the average root-mean-square deviation (RMSD) of the ligand) is less or equals 5Å as well as σ (standard deviation of RMSD of the ligand) is less or equals 0.5Å and another with all compounds for different trajectory segments.

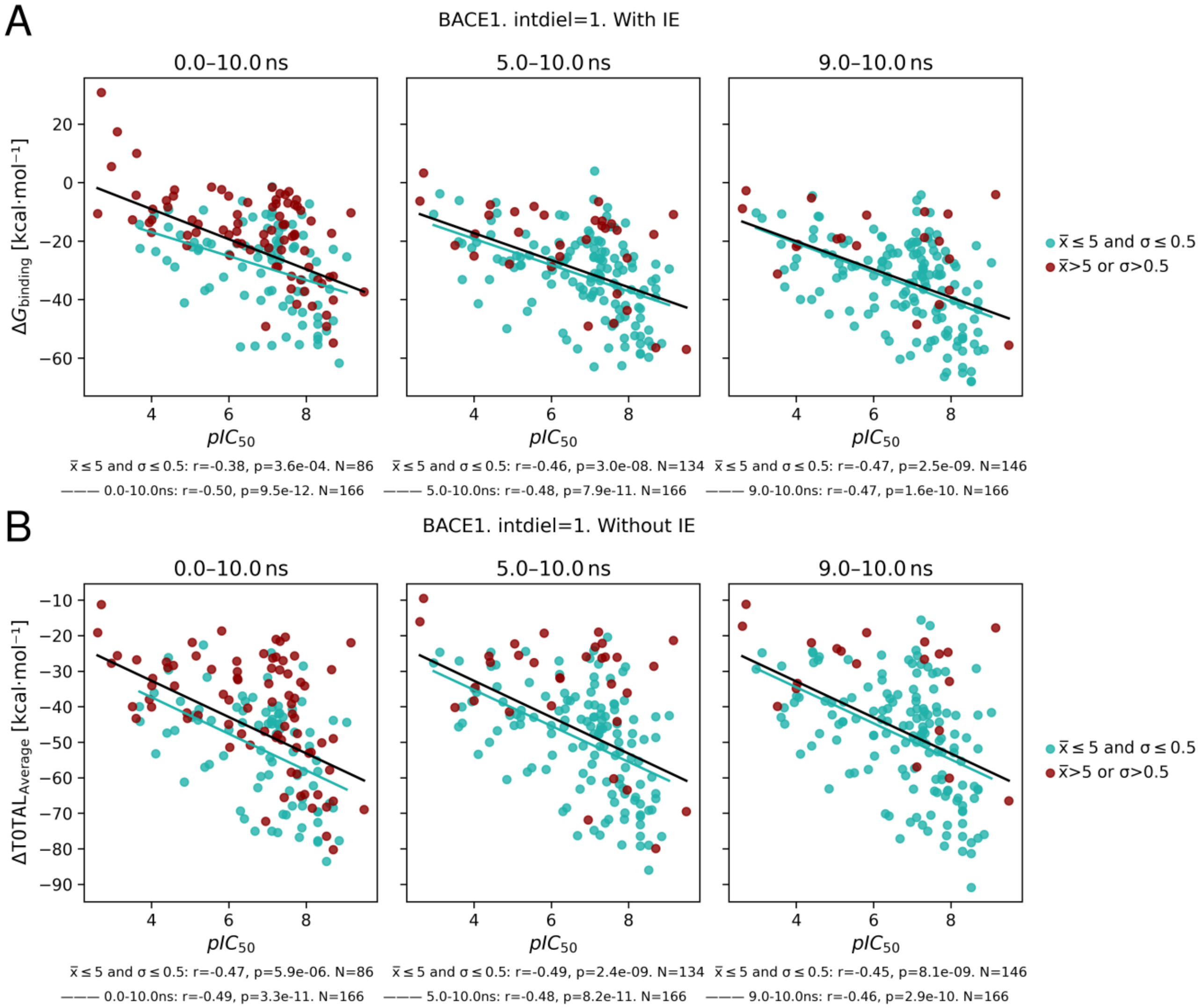

Figure S2. Correlation between experimental affinity values (pIC_{50}) of BACE1 dataset ligands and the calculated GBSA binding free energies taking into account interaction entropy (IE) term (**A)** and not (**B**) for different trajectory segments. The MM/GBSA internal dielectric constant (*intdiel*) was set to 1.0. Pearson correlation coefficient was calculated for each method separately for two groups of compounds, one where x̄ (the average root-mean-square deviation (RMSD) of the ligand) is less or equals 5Å as well as σ (standard deviation of RMSD of the ligand) is less or equals 0.5Å and another with all compounds for different trajectory segments.

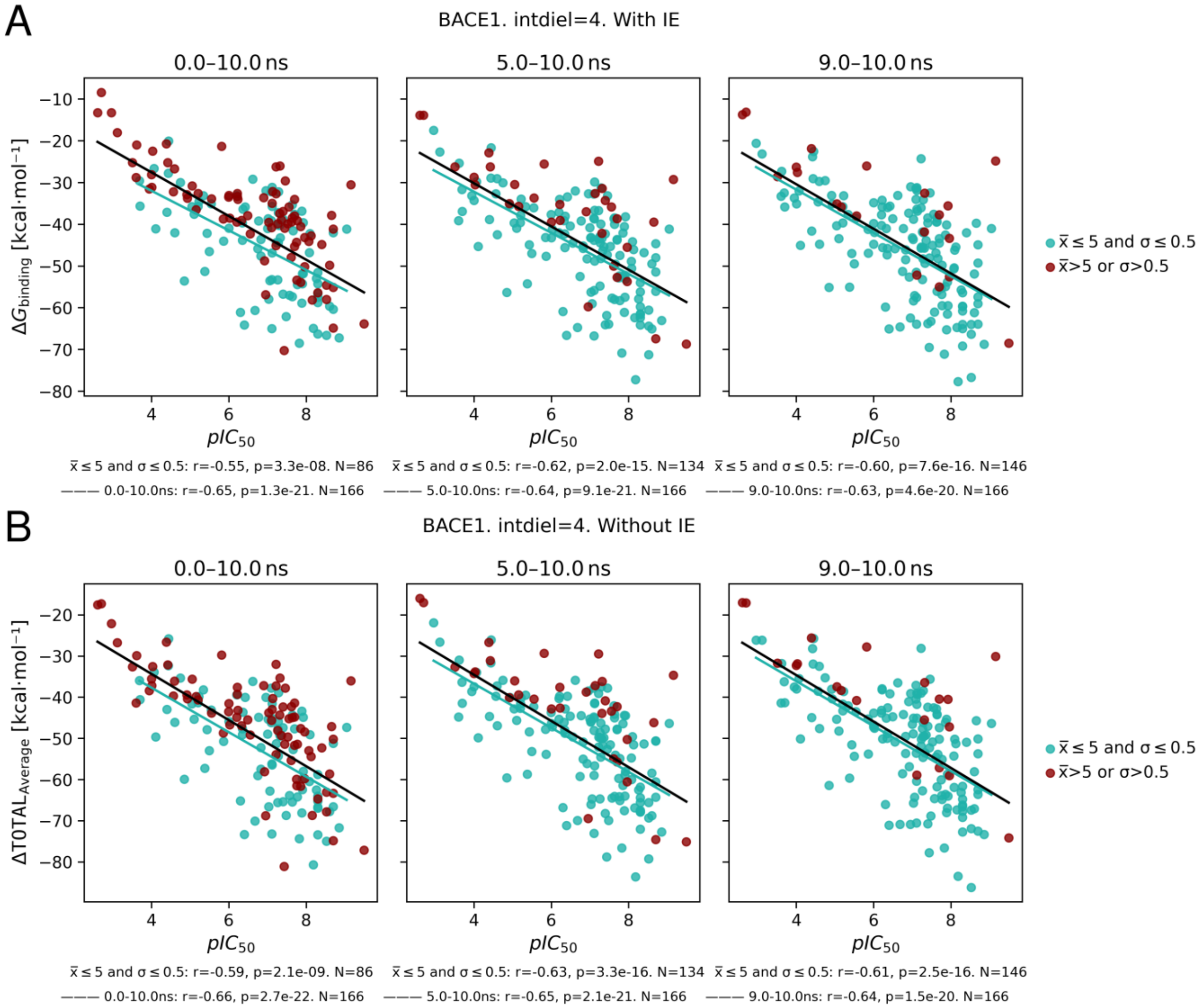

Figure S3. Correlation between experimental affinity values (pIC_{50}) of BACE1 dataset ligands and the calculated GBSA binding free energies taking into account interaction entropy (IE) term (**A)** and not (**B**) for different trajectory segments. The MM/GBSA internal dielectric constant (*intdiel*) was set to 4.0. Pearson correlation coefficient was calculated for each method separately for two groups of compounds, one where x̄ (the average root-mean-square deviation (RMSD) of the ligand) is less or equals 5Å as well as σ (standard deviation of RMSD of the ligand) is less or equals 0.5Å and another with all compounds for different trajectory segments.

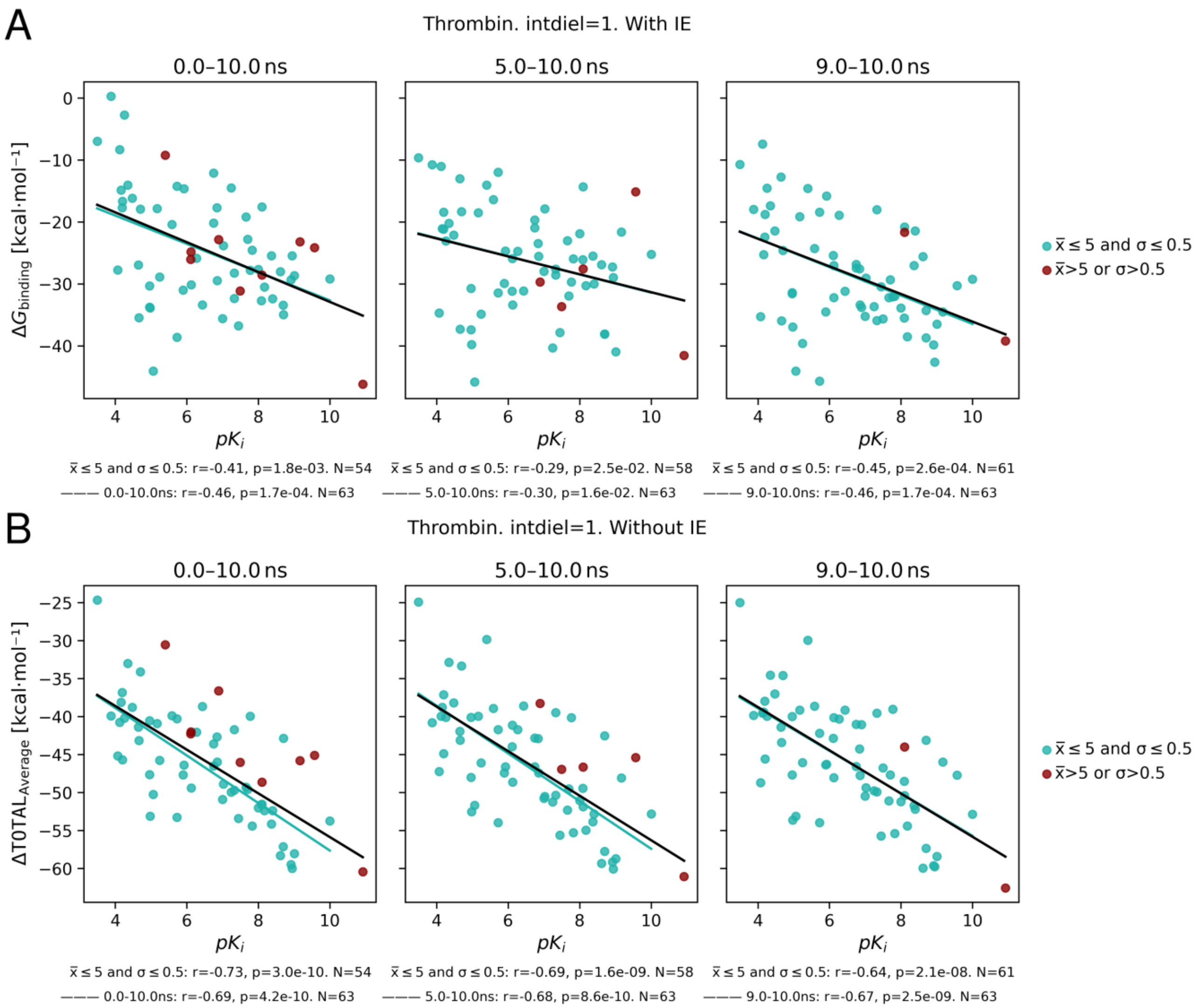

Figure S4. Correlation between experimental affinity values (pK_i) of Thrombin dataset ligands and the calculated GBSA binding free energies taking into account interaction entropy (IE) term (**A)** and not (**B**) for different trajectory segments. The MM/GBSA internal dielectric constant (*intdiel*) was set to 1.0. Pearson correlation coefficient was calculated for each method separately for two groups of compounds, one where $\bar{x}$ (the average root-mean-square deviation (RMSD) of the ligand) is less or equals 5Å as well as σ (standard deviation of RMSD of the ligand) is less or equals 0.5Å and another with all compounds for different trajectory segments.

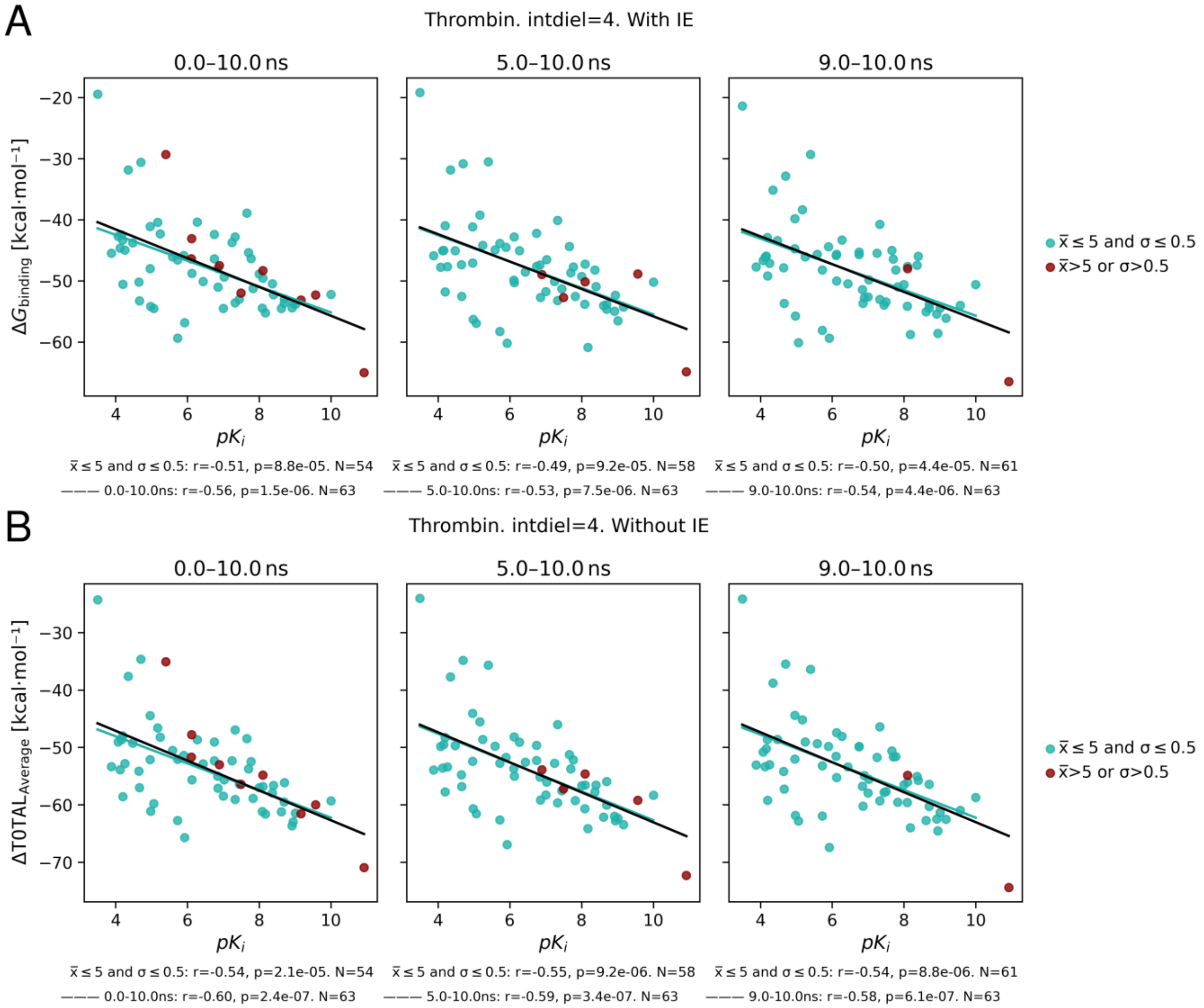

Figure S5. Correlation between experimental affinity values (pK_i) of Thrombin dataset ligands and the calculated GBSA binding free energies taking into account interaction entropy (IE) term (**A)** and not (**B**) for different trajectory segments. The MM/GBSA internal dielectric constant (*intdiel*) was set to 4.0. Pearson correlation coefficient was calculated for each method separately for two groups of compounds, one where $\bar{x}$ (the average root-mean-square deviation (RMSD) of the ligand) is less or equals 5Å as well as σ (standard deviation of RMSD of the ligand) is less or equals 0.5Å and another with all compounds for different trajectory segments.

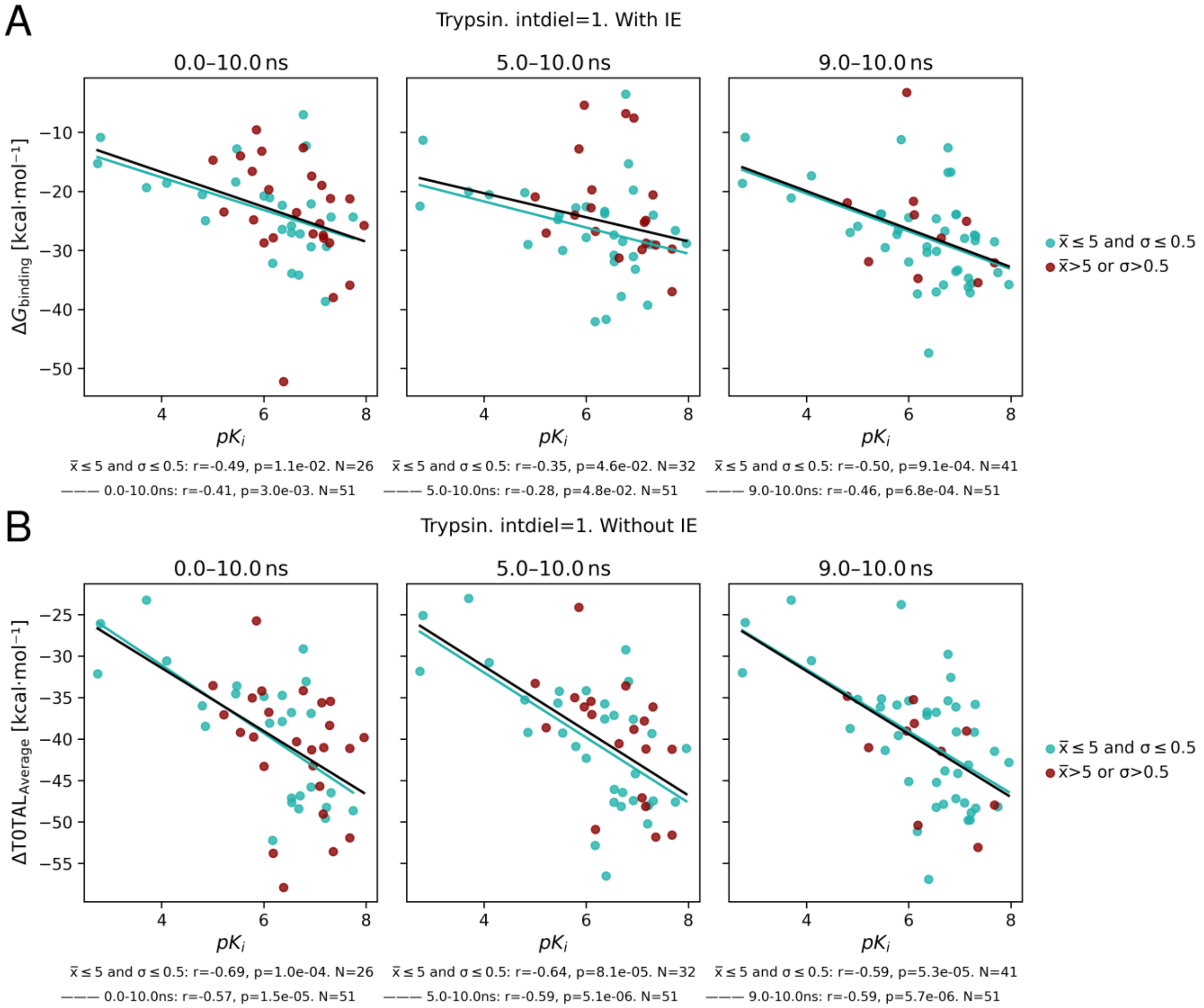

Figure S6. Correlation between experimental affinity values (pK_i) of Trypsin dataset ligands and the calculated GBSA binding free energies taking into account interaction entropy (IE) term (**A)** and not (**B**) for different trajectory segments. The MM/GBSA internal dielectric constant (*intdiel*) was set to 1.0. Pearson correlation coefficient was calculated for each method separately for two groups of compounds, one where x̄ (the average root-mean-square deviation (RMSD) of the ligand) is less or equals 5Å as well as σ (standard deviation of RMSD of the ligand) is less or equals 0.5Å and another with all compounds for different trajectory segments.

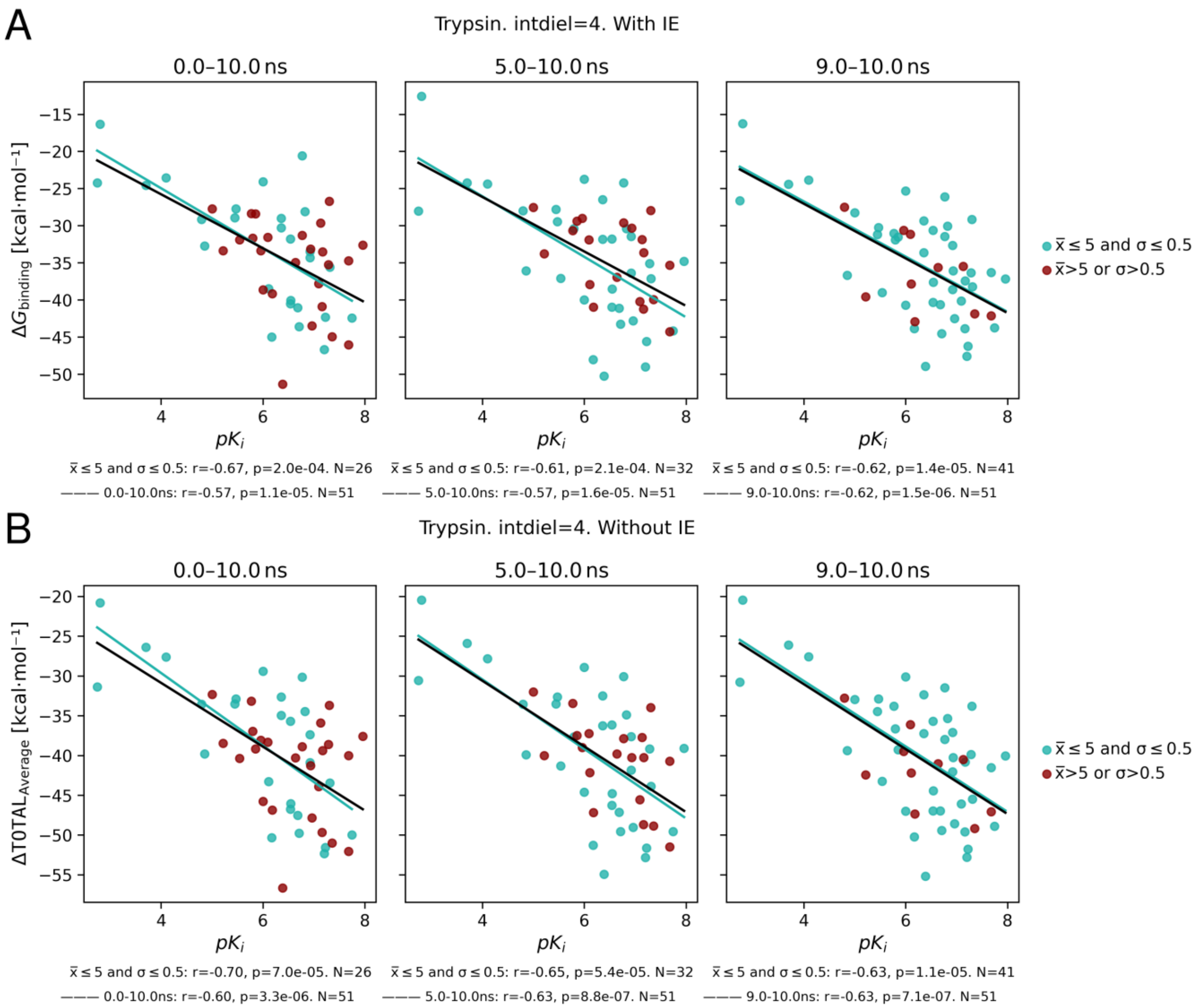

Figure S7. Correlation between experimental affinity values (pK_i) of Trypsin dataset ligands and the calculated GBSA binding free energies taking into account interaction entropy (IE) term (**A)** and not (**B**) for different trajectory segments. The MM/GBSA internal dielectric constant (intdiel) was set to 4.0. Pearson correlation coefficient was calculated for each method separately for two groups of compounds, one where x̄ (the average root-mean-square deviation (RMSD) of the ligand) is less or equals 5Å as well as σ (standard deviation of RMSD of the ligand) is less or equals 0.5Å and another with all compounds for different trajectory segments.

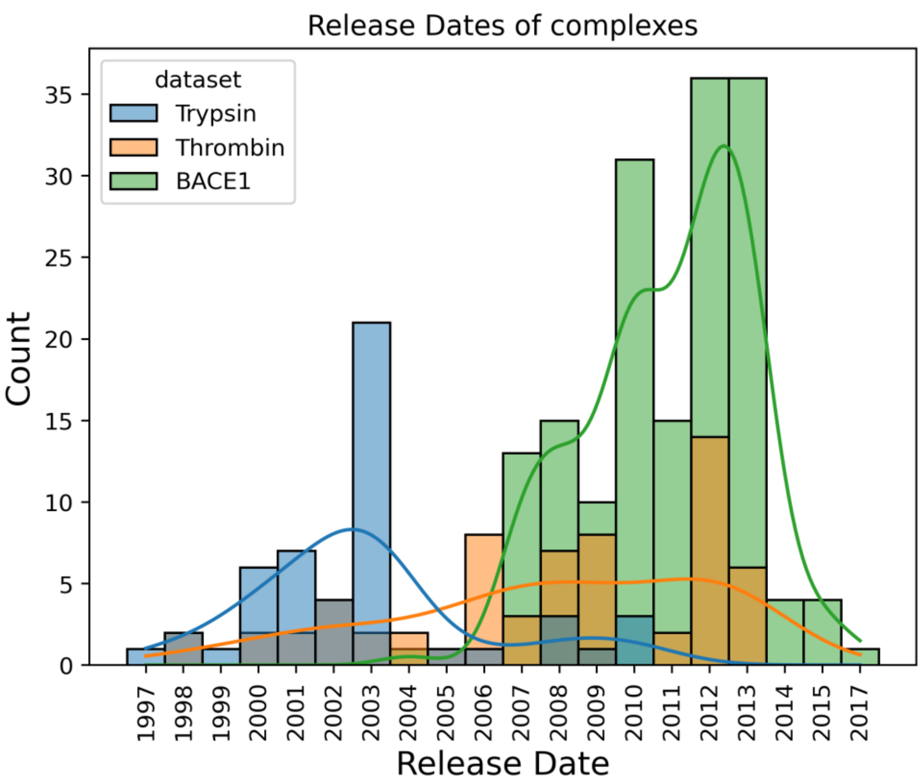

Figure S8. Release year histogram for BACE1, Thrombin and Trypsin datasets.

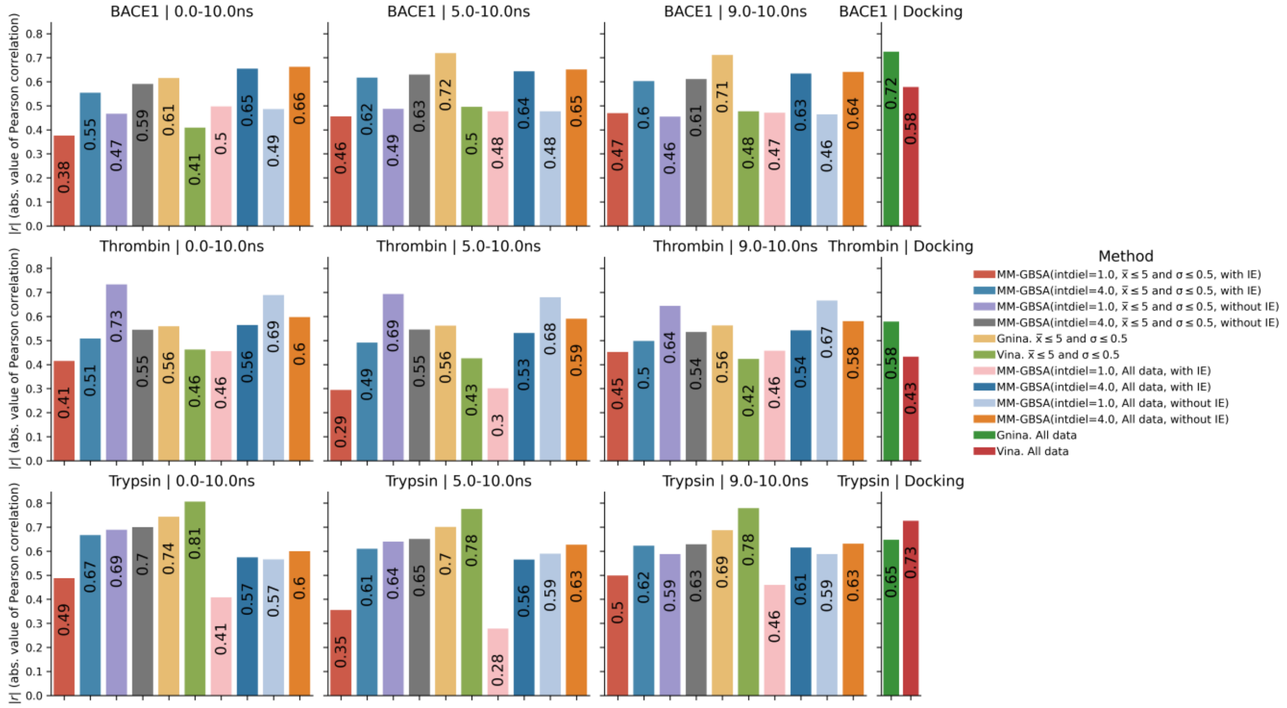

Figure S9 Correlation of calculated binding free energy and docking scores with experimental affinity for BACE1, thrombin and trypsin datasets and for different time frames. Pearson correlation coefficient was calculated for each method separately for two groups of compounds, one where x̄ (the average root-mean-square deviation (RMSD) of the ligand) is less or equals 5Å as well as σ (standard deviation of RMSD of the ligand) is less or equals 0.5Å and another with all compounds for different trajectory segments.

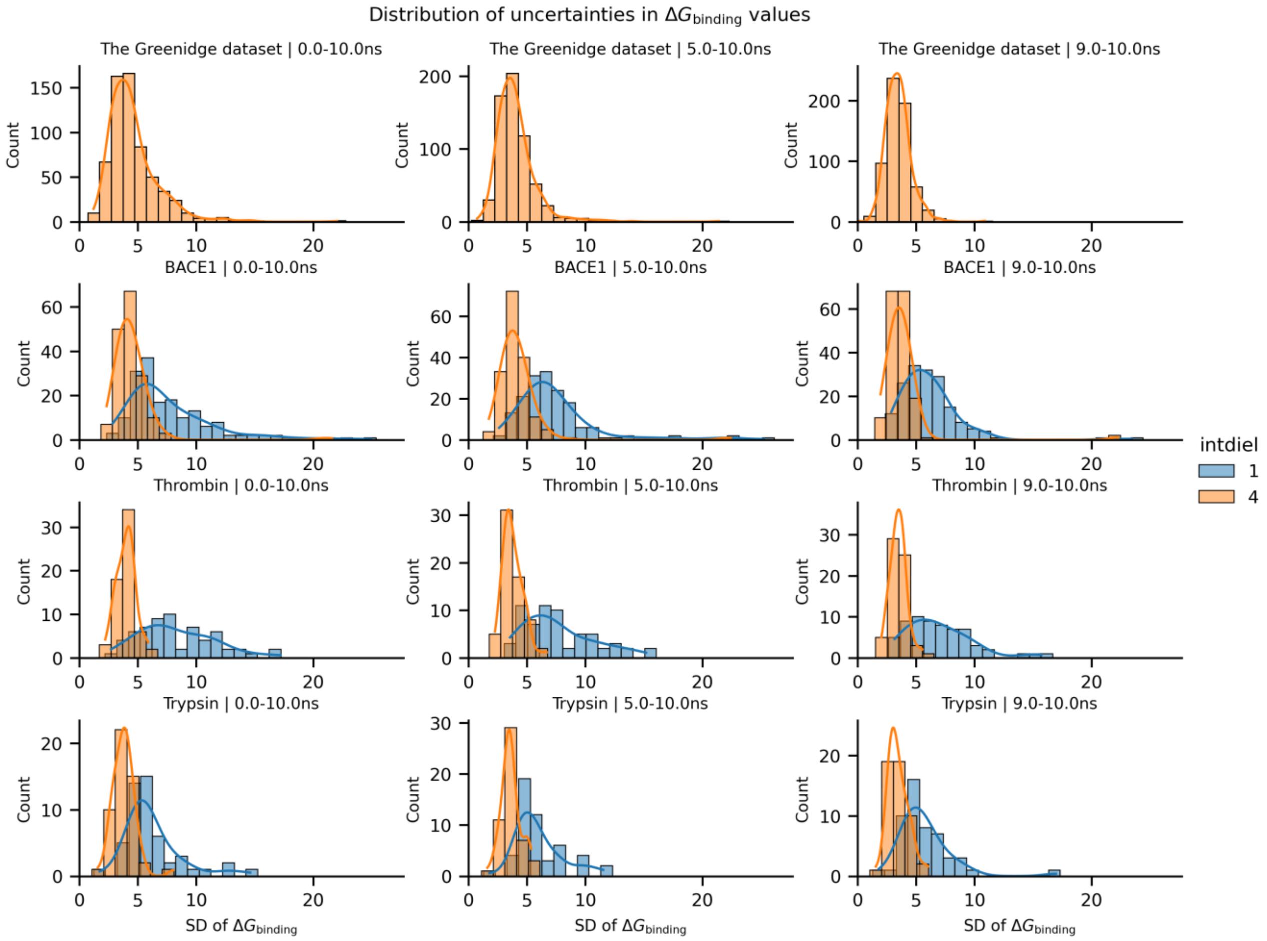

Figure S10. Distribution of errors of calculated binding free energy considering entropy for all four datasets, different intdiel values and time frames.

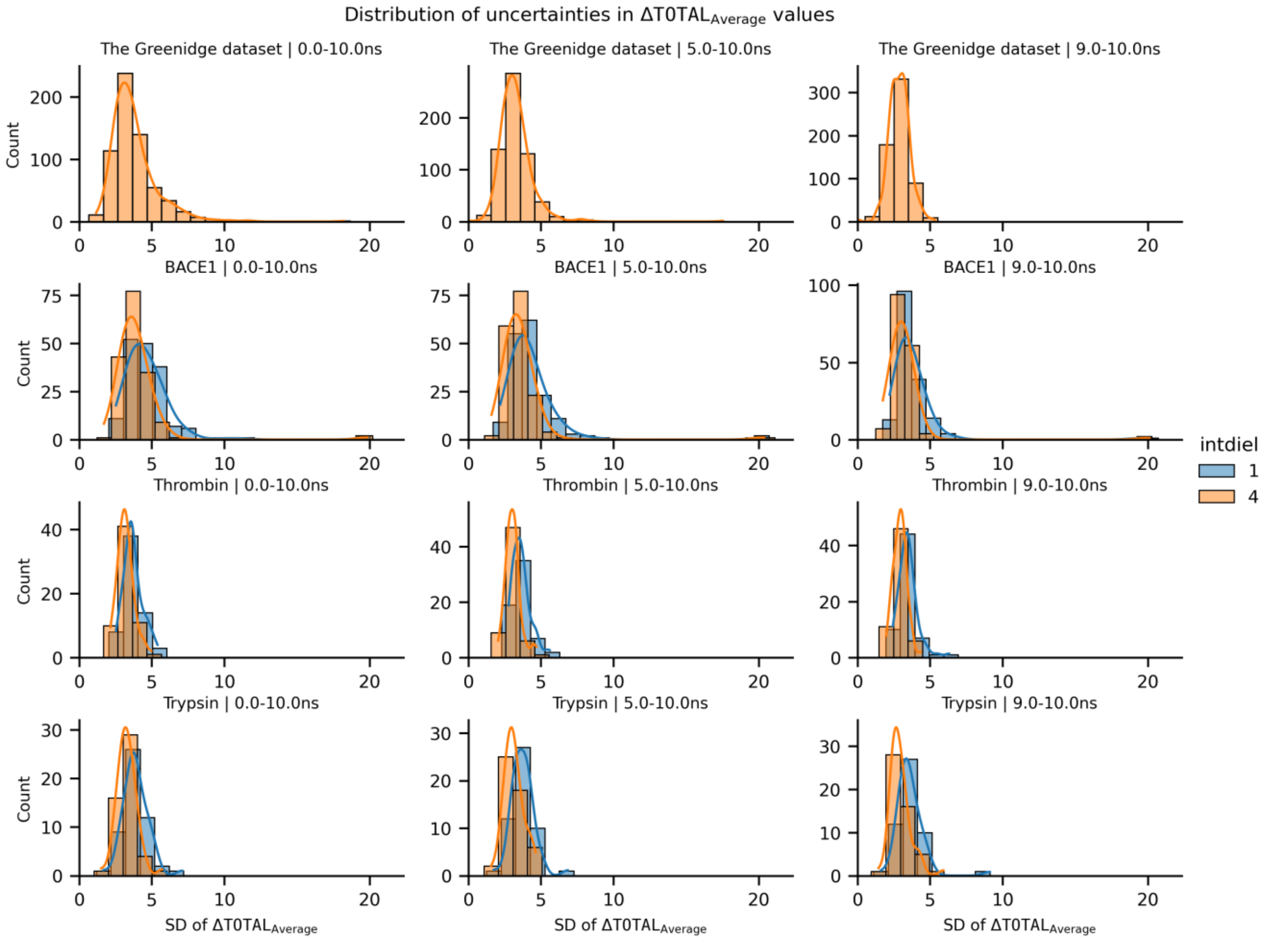

Figure S11. Distribution of standard deviations of calculated binding free energy disregarding entropy for all four datasets, different intdiel values and time frames.
